# Quorum Sensing Modulators as Antibiotic Alternatives in Animal Production: From Bacterial Signaling to Gut Health and Performance

**DOI:** 10.3390/vetsci13060507

**Published:** 2026-05-22

**Authors:** Chenxin Tang, Kehui Ouyang, Mingren Qu, Qinghua Qiu

**Affiliations:** Jiangxi Province Key Laboratory of Animal Nutrition and Feed, College of Animal Science and Technology, Jiangxi Agricultural University, Nanchang 330045, China

**Keywords:** animal feed, environmental sustainability, intestinal homeostasis, gut microbiota, production efficiency, quorum sensing, stress alleviation

## Abstract

In intensive animal production, the overuse of antibiotics has exacerbated bacterial antimicrobial resistance and environmental pollution. Together with gut microbiota dysbiosis and recurrent disease outbreaks, these challenges severely constrain the sector’s high-quality development. Quorum sensing (QS), a cell-density-dependent bacterial communication mechanism, can be modulated through agents that specifically inhibit or activate QS circuitry to regulate microbial community functions. Such QS modulators possess notable advantages, such as environmental benignity and high target specificity, and thus offer innovative strategies to decrease antibiotic reliance, enhance production efficiency, and reduce environmental emissions. This review examines QS modulators sourced from plants, microorganisms, animals, and synthetic processes, while highlighting key challenges such as environmental interference, resistance development, high costs, and the lack of standardized biosafety evaluations. Future research should focus on enhancing specificity, stability, affordability, and safety, with an emphasis on rational design, synergistic systems, improved manufacturing processes, and multi-target modulators. This review may provide a theoretical basis for translating QS-regulation technologies into farm-level applications, thereby advancing sustainable animal production and antibiotic-free husbandry.

## Simple Summary

The overuse of antibiotics in intensive farming contributes to antibiotic resistance, environmental pollution, and disruptions to microbial balance, posing significant risks to both animal and human health. Quorum sensing modulators offer a targeted, non-lethal alternative that minimizes resistance risk, yet research remains fragmented across sources and species. This review discusses both natural and synthetic quorum sensing modulators used in animal feed, their functions in gut health and production, and the current limitations such as unstable efficacy and high pricing. It also highlights potential areas for future research aimed at enhancing safety and practical application, presenting an environmentally friendly alternative to antibiotics in animal farming.

## 1. Introduction

Animal production is a critical industry for ensuring food safety and agricultural supply. Nonetheless, the chronic abuse of antibiotics in disease prevention and growth stimulation not only increased to spread drug resistant bacterial strains which were harmful to the human population but also resulted in the concentration of antibiotic resistance genes in animal waste thus contributing to environmental pollution [1]. At the same time, animal production faces numerous problems, including issues such as poor gut microbiota, difficulties in controlling bacterial diseases, inefficient feed digestion and absorption, as well as heat stress-induced intestinal barrier damage, oxidative stress, and immunosuppression, all of which, under the direct influence of such factors, determine the limitation of the increase in production efficiency and the quality of products [2,3]. These challenges add complexity to achieving the objectives of animal farming, which focus on enhancing quality and efficiency while promoting green and sustainable development. Quorum sensing (QS) is a cell-density-dependent communication system commonly found in bacteria [4]. It utilizes autoinducer signals to coordinate collective behaviors, playing a crucial role in regulating the secretion of various virulence factors and the entire process of biofilm formation [4,5]. As a result, QS serves as a key regulator of bacterial pathogenicity and a central target for developing novel antibacterial strategies. QS modulators regulate microbial community functions by inhibiting or activating bacterial QS systems without directly killing bacteria, thereby reducing the risk of antibiotic resistance development [6,7]. Notably, many QS modulators also have the effect of inhibiting virulence expression and not inducing drug resistance, making them ideal candidates for achieving resistance reduction and substitution [8]. However, its disadvantages are a lack of uniform efficacy, vulnerability to the development of resistance, and difficulty in mass production. This review summarizes the application progress of QS modulators from diverse sources in animal production based on QS signaling molecular regulation mechanisms. It integrates fragmented research findings and establishes an application framework for QS modulators in animal production, while analyzing current challenges and innovative directions. This provides a comprehensive reference for technological transformation and industrial application of QS modulators in animal production.

## 2. Types of QS Signaling Molecules

Depending upon the differences in signaling molecules, the QS forms are mainly of three categories. The two-component QS system of Gram-positive bacteria employs autoinducer peptides for communication. It consists of a transmembrane receptor histidine protein kinase and a homologous cytoplasmic response regulator, which constitute signal transduction activity undertaken by a phosphorylation cascade to control the behavior of bacterial populations [9]. The LuxS/AI-2 QS system, shared by Gram-positive and Gram-negative bacteria, is the most widely distributed QS system. Its signaling molecule is autoinducer-2 (AI-2) whose production mostly depends on the LuxS enzyme and is also produced via the phosphoenolpyruvate isomerase pathway [10,11]. The signaling molecules of the LuxI/LuxR QS system in Gram-negative bacteria are acyl-homoserine lactones (AHLs), which are synthesized by LuxI-type proteins and diffused into the extracellular space [12,13]. When concentrations reach a threshold, AHLs re-enter the cell, where they bind to LuxR-type proteins to form complexes. These complexes can specifically activate or inhibit the transcription of target genes, thereby regulating the expression of related genes [12,13]. It is important to mention that the functions of the QS system presented above are not fixed and are greatly modulated by environmental conditions. High temperatures can modify the synthesis, secretion, and receptor binding of QS signaling molecule, and finally disrupt interbacterial communication and the homeostasis between the host and the microbial community under heat stress conditions [14].

## 3. Quorum Sensing Inhibitors in Animal Production

**Figure 1:**
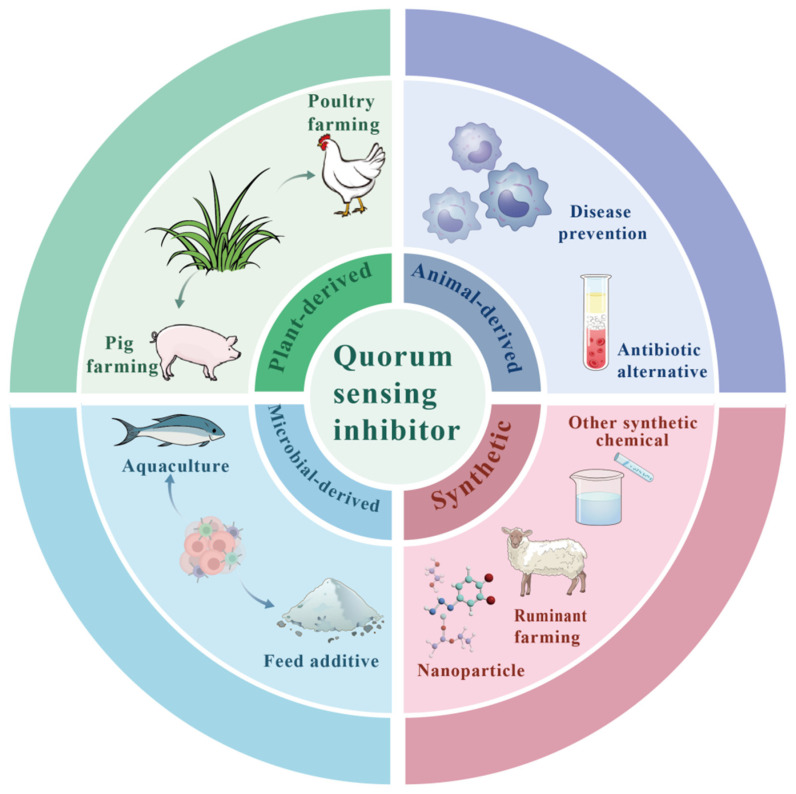
The potential applications of quorum sensing inhibitors in animal production.

Quorum sensing inhibitors (QSI) are biologically or chemically active substances capable of specifically disrupting microbial QS systems. They are able to prevent or disrupt pathogenic behaviors in the expression of virulence factors, biofilm formation by inhibiting the synthesis of signal molecules, active degradation or scavenging of signal molecules through enzymatic activity or competitively binding to signal receptors [7,8]. Due to their safety and targeted regulatory properties, they have emerged as important candidate substances for replacing antibiotics and controlling pathogenic bacteria in animal production [8]. Nevertheless, the risk of bacterial resistance to long-term QSI use requires further investigation. QSI can be classified into naturally occurring inhibitors and synthetic inhibitors based on their sources. Naturally occurring QSI may be further grouped into three large categories, which include plant-derived, microbial-derived, and animal-derived. Figure 1 summarizes the potential uses of QSI in animal production.

### 3.1. Plant-Derived QSI

Plant-based QSI is an extract that is widely available, highly biocompatible, and has comparatively developed extraction procedures, and is most commonly used in animal production [15]. It contains polyphenols, terpenoids, aldehydes, and similar substances as its main active components and they are summarized in Table 1. In poultry production, Deryabin et al. [16] found that the dietary supplementation of quercetin, vanillin and umbelliferone either singularly or combined reduced intestinal inflammation and increased production efficiency in broilers. These compounds have anti-inflammatory and antioxidant effects and have the potential to improve the composition and structure of the gut microbiota; thus, they have the theoretical potential to mitigate intestinal oxidative stress and inflammatory reactions due to heat stress in broiler chicken living in the high-temperature environment. Simultaneously, it is reported that chlorogenic acid (CA) can effectively prevent intestinal mucosal damage in laying hens subjected to acute heat stress by inhibiting the QS systems of pathogenic bacteria, suppressing intestinal inflammation, and modulating gut microbiota composition [17]. Furthermore, plant-derived monoterpenes, such as (-)-*α*-pinene, have been found to reduce the colonization of *Campylobacter jejuni* in broilers without adversely affecting growth performance, showing promising potential as antibiotic-free feed additives [18]. In swine production, addressing disease-induced mortality in piglets is a major factor driving antibiotic use. CA has been shown to lower diarrhea incidence, improve weight gain, and relieve oxidative stress and inflammation in weaned piglets [19]. Resveratrol is also known to enhance the intestinal microenvironment of suckling piglets when exposed to high-temperature heat stress [20]. Subsequent studies by Wei et al. [21] found that the combination of curcumin and resveratrol produces synergistic effects; such a combination not only helps to curb the generation of oxidative damage on its own but also enhances antioxidant defense mechanisms of the body, which is even more comprehensive than the action of curcumin alone through the single pathway regulation. Notably, environmental factors (pH, temperature, oxygen) significantly compromise the stability of QS modulators in practice. Combining plant-derived QSI with other chemical or physical approaches can enhance its inhibitory efficacy. For example, under photocatalytic conditions, the inhibitory effect of curcumin can be amplified, suggesting that such plant-based QSI combinations under light-assisted methods represent a promising direction for the development of plant-derived QSI applications [22]. For *Streptococcus suis*, the bacterium capable of causing septicemia in piglets, paeoniflorin has been found to inhibit its LuxS/AI-2 QS system and to reduce adhesion-associated virulence, thereby improving piglet survival [23]. In the context of meat preservation, cinnamaldehyde and eugenol have been observed to extend the shelf life of chilled pork by inhibiting *Escherichia coli* AI-2 signaling and reducing biofilm formation, offering a novel strategy for green meat preservation [24]. In aquaculture, methyl gallate extracted from *Camellia nitidissima* Chi flowers has been shown to inhibit the virulence and biofilm formation of *Aeromonas hydrophila*, thereby reducing mortality rates and losses associated with gill rot and enteritis in fish [25]. *Ocimum sanctum* acts as a QSI by targeting bacterial communication pathways, reducing pathogenicity without disrupting normal bacterial growth. It shows potential for managing antibiotic-resistant infections in aquaculture [26]. Similarly, Gu et al. [27] demonstrated that artemisinin activates the nuclear factor kappa B signaling pathway in fish intestinal tract, alleviates intestinal inflammation and tissue damage caused by sluggish *Edwardsiella tarda*, thereby optimizing gut microbiota structure and improving physiological health. Notably, natural astragalus polysaccharides have been proven to alleviate oxidative stress and inflammation in aquatic animals under heat stress by regulating key signaling pathways related to antioxidation, inflammation, and immune responses. These mechanisms effectively reduce oxidative damage and inflammatory reactions, thereby alleviating stress in aquatic animals [28]. Preparations of medicinal plants, composite medicinal plant preparations, are important in animal production. For example, *Ficus carica* L. and *Perilla frutescens* exhibit significant QSI activity and can serve as natural alternatives for combating infections and multidrug resistance [29]. Plant essential oil preparations have been beneficial in terms of enhancing average daily gain and feed conversion ratio, ammonia nitrogen levels within the rumen, as well as milk and meat quality [30].

### 3.2. Microbial-Derived QSI

Regarding microbial-derived QSI, one can distinguish enzymes, peptides/bacteriocins, secondary metabolites, and biosurfactants, and the corresponding substances are available in Table 1. Enzymes possessing quorum quenching activity, such as lactonases, acyltransferases, and oxidoreductases, were originally discovered in the genus *Bacillus* [47]. Notable examples include the YtnP lactonase from *Bacillus velezensis* D-18, which suppresses *Vibrio anguillarum* QS pathways. Its dual antibacterial and quorum quenching properties position it as a safe probiotic candidate for vibriosis management in aquaculture [33]. Similarly, the AiiA lactonease secreted by *Bacillus thuringiensis* can specifically degrade the AHL QS signaling molecules of pathogenic bacteria, thereby improving broiler production performance and regulating intestinal microbial communities, thus providing a bioactive solution for pathogen control in livestock and poultry farming [34]. Additionally, Jha et al. [35] reported that cyclo(L-Phe-L-Pro) inhibit *Salmonella typhi* virulence and biofilm formation with high biosafety, highlighting their potential in antibiotic-free poultry farming. Additionally, the cyclic lipopeptide surfactin from *Bacillus subtilis* 6D1 was found to impede the Agr QS pathway in multidrug-resistant *Staphylococcus aureus*, thereby hindering biofilm formation and bolstering host immunity [36]. Practically, dietary supplementation with lipopeptide QSIs in piglets presents a novel approach to mitigating gut dysbiosis and enhancing stress resilience. The screening of secondary metabolites remains an extensive and ongoing area of research, frequently applied in livestock and poultry feed or biological control applications. For example, Devi et al. [37] first reported the inhibitory activity of *Bacillus subtilis* R-18 against the LuxI/LuxR QS system and identified its active metabolite as 2,4-di-tert-butylphenol. Similarly, indole metabolites from *Pseudomonas* can block QS by competing for AHL receptors, thereby reducing virulence [38]. Despite the fact that studies on the last category of biological surfactants are small yet active and promising. Lipopeptide biosurfactants identified from coral-associated *Bacillus* species exhibit excellent anti-biofilm activity, offering candidate molecules for marine green antifouling [48]. Moreover, some fungi secrete QS-inhibitory compounds. Specifically, the medicinal fungus *Phellinus igniarius* extracts are found to produce QSI [49]. Nouh et al. [39] have shown that *Penicillium oxalicum* AUMC 14898 (*Opontia ficus-indica*) endophytic fungus has anti-QS potential. Production of tannic acid by it prevents QS signal transduction and expression of biofilm-related genes in *Pseudomonas aeruginosa* [39]. This approach overcomes the resource constraints that go with direct plant harvesting that forms a basis for fungal QSI development and use in animal farming. Despite these advances, the precise mechanisms and molecular targets of many microbial QSI remain elusive. For example, while Gregatins can inhibit QS in *Pseudomonas aeruginosa*, they suppress only specific pathways rather than blocking the system entirely [50]. Similarly, Paecilomycone inhibits QS in *Pseudomonas aeruginosa* through multi-node suppression [51].

### 3.3. Animal-Derived QSI

Some of the QSI occur in animal tissues and cells and the corresponding substances are listed in Table 1. For example, ground beef extract also prevents AI-2 signaling activity and has an impact on the survival and virulence of *Escherichia coli* O157:H7, advertising the expression of virulence-related genes, including *yadK* and *hhA* [40]. This offers a new direction of QS inhibition on the management of this pathogen in meat processing. Notably, research indicates that the majority of animals known to produce QSI belong to marine ecosystems. Sponge-derived ilimaquinone is also anti-biofilm active against Gram-negative bacteria, which is a possible alternative to antibiotics in the production facility [41]. Payam et al. [42] discovered that sea cucumber saponins could inhibit binding of AHL signaling molecules to receptors, biofilm formation as well as virulence expression of *Aeromonas hydrophila* at sub-inhibitory levels, a green control method of *Aeromonas hydrophila* in aquaculture. Similarly, the peptide SF, screened from the myosin of *Penaeus vannamei*, possesses QS-inhibitory capabilities; it inhibits the QS signaling pathway of *Vibrio parahaemolyticus* and interferes with bacterial adhesion and aggregation, thereby reducing bacterial motility and infection risk [43]. Another study provided the first confirmation that solenopsin A in the venom of the red imported fire ant functions as a natural QSI; these compounds exhibit antivirulence properties without bactericidal effects, thus avoiding residue accumulation during breeding processes [44]. Notably, melittin has been demonstrated to possess triple activities against biofilms, bacteria, and QS, offering a new perspective for developing novel antibiotic alternatives [45]. Propolis was also a possible QSI, and Tamfu et al. [46] isolated and identified cycloartane-type triterpene acid in propolis that can inhibit QS in several bacterial species, explaining new prospects in the development of the propolis as a natural supplement in animal production. Moreover, the synergistic use of animal-based QSI and other ones may be surprisingly effective. For example, nanoemulsions (incorporating casein, lecithin, etc.) encapsulating carvacrol, citral, and eugenol serve as edible coatings for pork, effectively preserving meat flavor and nutritional quality [52].

### 3.4. Nanoparticle QSI

Nanoparticles are a class of materials with sizes of 1–100 nm and possessing unique chemical and physical properties. Some of these nanoparticles can act as QSI; their inhibitory effect on QS depends on factors such as the type of nanoparticle, the synthesis method, and the active concentration, among others [53]. Integrating nanoparticle-based QSI delivery systems with metagenomic and metabolomic platforms enables real-time surveillance of QSI biodistribution and the dynamic fluctuations of gut microbial QS signals. This synergistic approach establishes a technical foundation for engineering multi-targeted nanoparticle QSI characterized by enhanced specificity and reduced cytotoxicity. QSI based on metal or metal oxide nanoparticles represents a well-studied class of therapeutic agents. Wang et al. [54] designed a copper nanoparticle system coated with erythrocyte and platelet membranes to achieve dual functions: broad-spectrum bactericidal activity and QS inhibition. The bioinspired coating does not only give a bacterial power to the nanoparticles but also possesses action by modifying the microenvironment of the bacteria. Khan et al. [55] developed two distinct zinc oxide nanospikes capable of inhibiting QS across multiple pathogenic strains while attenuating the secretion of virulence factors such as pyocyanin and rhamnolipids. Furthermore, green-synthesized silver nanoparticles have been demonstrated to disrupt both QS and biofilm formation in *Pseudomonas aeruginosa* [56]. The sum of these studies asserts the shortcoming of the poor biocompatibility of the conventional antimicrobial method. While enhancing antimicrobial efficacy, they provide novel nanoscale technological approaches for controlling pathogenic bacteria in animal production. In a related context, zinc/carbon nanomaterials have been shown to repress antibiotic resistance genes during the composting of cattle manure by modulating QS and microbial community dynamics, providing an eco-friendly technological pathway for controlling resistance pollution in livestock waste [57]. Another type is nanoparticle QSI using nanoparticles as a delivery system, where chitosan-based nanoformulations loaded with natural products hold greater translational potential than metallic nanoparticles due to their excellent biocompatibility. For example, chitosan-silver nanocomposites derived from maggot-source chitosan demonstrate high-efficiency, low-toxicity antimicrobial activity against fish pathogens, emerging as superior green candidates for aquaculture compared with either chitosan or silver nanoparticles alone [58]. Liposome-encapsulated natural products also play a substantial role in animal production. Resveratrol-loaded liposomal nanocarriers have been shown to enhance growth performance, restore productivity, and mitigate oxidative damage in heat-stressed broilers [32]. Najafi et al. [59] prepared ellagic acid-loaded liposomes, which were found to enhance sperm mitochondrial activity after freeze–thaw cycles, fully meeting the requirements for improving the preservation and reproductive efficiency of livestock and poultry semen. Simultaneously, soy lecithin-derived nanoliposome technology and lycopene-loaded nanoliposome technology can also enhance sperm motility, offering viable solutions for improving semen cryopreservation efficiency and subsequent artificial insemination fertilization rates [60,61]. In the aquaculture sector, Besharat et al. [62] confirmed that nano-liposomal encapsulation of astaxanthin improves hematological parameters, immune response, and antioxidant capacity in rainbow trout, validating the high-efficiency application of natural pigment nanocarriers in aquatic feeds. Notably, the QS mechanism can also be achieved through surfactant competition in non-living systems such as emulsion droplet clusters. The discovery of this phenomenon may provide insights into the design of novel biomimetic QS nanomaterials [63]. Nevertheless, the deployment of such synthetic modulators—particularly nanoparticle-based formulations in animal production contexts—necessitates rigorous biosafety assessments to minimize potential cytotoxicity and adverse environmental impacts associated with these compounds.

### 3.5. Other Synthetic QSI

Beyond nanoparticle-assisted QSI, a major advancement has been achieved on the design and use of pure synthetic QSI. Helmy et al. [64] demonstrated that QSI-5, a synthetically derived AI-2 QSI, specifically blocks the LuxS/AI-2 signaling system in avian pathogenic *Escherichia coli*, thereby reducing mortality rates in livestock and poultry. Since the high-temperature season coincides with the peak incidence of pathogens such as avian pathogenic *Escherichia coli*, such QSIs can serve as an alternative to antibiotics to enhance the disease resistance of chicken flocks during hot weather. Synthetic cannabinoid HU-210 also has the potential of lowering the pathogenicity of *Vibrio harveyi* by disrupting its QS pathway hence regulating aquatic diseases [65]. Moreover, the luteolin-borneol complex that was created by Zhou et al. [66] proved to be effective in the treatment of nervous necrosis disease in grouper juveniles, which provides a new approach to the development of viral disease management in aquaculture. To address the severe threat of vibriosis in aquaculture, researchers formulated and produced thiophenone compound TF310 [67]. The compound is capable of interfering with the QS mechanism of a range of pathogenic *Vibrio* species, therefore offers effective protection to sterile brine shrimp larvae and cures the multiple types of *Vibrio*-induced aquatic diseases [67]. In addition, to address the problem of cyanobacterial blooms, which is very widespread, scientists discovered a new QS molecule, 3-OH-C4-HSL, and then developed a direct inhibitor, dihydro-3-amino-2(3H)-furanone, based on its structure [68]. Similarly, synthetic methyl anthranilate was found to disrupt the biofilm architecture of *Streptococcus suis* and may serve as a novel therapeutic adjuvant for preventing porcine sepsis in piglets [69]. Additionally, synthetic D-ribose has been shown to modulate ruminal QS in Hu sheep, enhancing their growth efficiency [70]. Enhanced antioxidant capacity is particularly important for counteracting oxidative damage caused by heat stress. In addition, heated drinking water was also discovered to enhance the growth performance of male Hu sheep, regulating rumen QS and metabolites and also increasing serum antioxidant capacity [71]. This suggests that temperature changes can influence rumen microbial function and host production performance via the QS pathway. Currently, multiple derivatives have been demonstrated to significantly inhibit the QS systems of various pathogenic bacteria, such as N-acyl-homoserine lactone triazoles, sulfonamides, and sulfonylurea derivatives, providing new strategies for drug development targeting related diseases [72]. Nayak et al. [73] further demonstrated that synthetic chalcone derivatives significantly inhibit the QS system of *Salmonella*, enabling effective control of pathogens in poultry meat. To address and purify issues such as manure waste, livestock and poultry wastewater, and aquaculture effluent during production processes, numerous synthetic QSI compounds have replaced chemical agents like antibiotics in aquaculture environmental management. Previous studies have demonstrated that synthetic 5,6-dimethyl-2-amino-benzofuranone derivatives can mitigate biofilm contamination [74]. It is also noteworthy that Zhang et al. [75] indicated that synthetic QSI serves as an effective strategy to reduce the abundance of human bacterial pathogens in soil, significantly decreasing both intraspecific and interspecific conjugation frequencies among bacteria. This provides a safety rationale for promoting the use of synthetic inhibitors in livestock environmental management.

## 4. Quorum Sensing Activator in Animal Production

Quorum sensing activators (QSA) are substances that are able to stimulate, sensitize, or expedite bacterial QS signaling pathways, thereby upregulating the QS-regulated phenotypes in bacteria [76]. In comparison to QSI studies, those regarding QSA are rather few, but they demonstrate considerable potential and benefits in the improvement of the functionality of beneficial bacteria. Mannan oligosaccharides being a representative QSA are able to generate mannooligosaccharide selenium complexes with selenium [77]. This compound not only augments the proportional number of good bacteria within the intestines of the weaned piglets, but also the functionality of intestinal mucosal immunities, and at the same time augments the rate of feed-digestion and absorption [77]. Likewise fructooligosaccharides are also said to optimize the intestinal microbiota of calves leading to those animals showing more nutrient digestibility as well as a superior growth performance [78]. Curcumin, an antibiotic alternative in broiler chickens, also stimulates the increase in good bacteria like *Lactobacillus* and *Bifidobacterium* and depresses the pathogenic activity hence decreasing the mortality rates and diarrhea levels [31]. Furanone, a rumen feed additive, also stimulates microbial QS in the rumen, controls microbial concentration, and hence improves the growth performance of Hu sheep [79]. In particular, supplementation with 4-hydroxy-2,5-dimethyl-3(2H)-furanone enhanced serum antioxidant capacity and nutrient digestibility in Hu sheep, which promoted rumen microbial density and biofilm formation [80]. This dose-effect illustrates once again the possibility of furanone-derived QSA in the production of ruminants. Research has also found that dietary bile acids supplementation enhanced rumen fermentation and growth performance in culled ewes by upregulating AI-2-mediated QS and facilitating biofilm formation within the rumen microbiota [81]. Furthermore, studies indicate that adding AHL analogs activates the QS pathway within bioflocs, accelerating their maturation and stabilizing metabolic processes, making it an effective means to enhance aquaculture efficiency [82]. QSA is also involved in the environmental management of breeding thus properly mitigating the pollution of water by wastewater by increasing biofilms, nitrogen metabolism and other techniques. Li et al. [83] demonstrated that supplementation of 3-oxo-C14-HSL into wastewater enhances the expression of the genes of nitrogen metabolism and QS-related ones into biofilms, stimulates the enrichment of denitrifying bacteria, and helps to solve the biofilm formation and structural stability problem in the treatment of aquaculture wastewater. Additionally, C6-HSL and N-3-oxo-C8-HSL are also confirmed to stimulate the QS pathways and improve denitrification performance [84]. To address challenges such as the difficulty of settling microalgae in livestock manure effluent, a research team found that C12-HSL was capable of inducing *Chlorella* to express aromatic proteins and subsequently cause bioflocculation and improved solid–liquid recovery [85]. It is worth mentioning that the addition of AHL analogs to wastewater containing nano-Ag can efficiently eliminate the pollutants without altering the stability of the sludge [76]. Exogenous surveillance of AHL-like QSA through targeted addition has the potential to provoke the QS system of beneficial bacteria in water and manure treatment facilities. This approach can optimize microbial communities, improve environmental pollution management, and enhance the efficacy of aquaculture systems. The application of QSA fills the gap in QS regulation for positively optimizing microbial communities, serving as a crucial tool for achieving enhanced aquaculture quality, increased efficiency, and environmental sustainability. The inverse of this is that although QSA has tremendous prospects in regulating useful microbiomes, their use is at an early stage. One of the main weaknesses is that their regulatory mechanisms are undetermined and there are no standard guidelines for dosage. Misuse of activation can interfere with the balance of microbial communities or stimulate the abnormal metabolic processes in animals. Thus, the clarity of the specific action mechanism, the most appropriate dosage levels, and the long-term safety profiles should be clarified before they are used on large-scale animal production.

## 5. Challenges and Future Perspectives

Despite the wide application perspectives of the QS modulators in animal production, there have been many challenges to large-scale application and technological transformation today. The robustness of QS modulators is more than sensitive to changes in agricultural conditions, high-temperature environments, physiological positions of animals, and the structure of microbiota living on the gut. The identical modulator can be found to have large improvements and repudiations in its performance in various farming conditions or types of animals, resulting in a decrease in bioavailability. To mitigate this variability, there will be the use of microencapsulation or a targeted release vehicle to contain the use of QS modulators to site selective release within the gut in order to eliminate degradation within the environment. Furthermore, developing context-specific modulators tailored to the unique microbial ecology and physiological traits of target animals could also improve the consistency and reliability of QS-based interventions. Moreover, a prolonged mono-treatment of a specific group of QSI may result in the adaptive mutations of bacteria. Higher bacterial resistance levels with synthetic furanone analogs have already been reported by other people and hence the risk of antimicrobial resistance spreading should be placed on the radar [86]. This resistance can occur because of gene mutation in the QS receptor or the large expression of efflux pump that might slowly erode the efficacy of QSI as time progresses, and high temperature may accelerate this process. Cost constraints and technical barriers in large-scale production also pose challenges for application. For example, microbial-derived QSI suffer from low yields and complex purification processes, while chemically synthesized QSI involve cumbersome synthetic steps and high raw material costs. Plant-derived QSI exhibit limited extraction rates and are significantly influenced by raw material origin and quality [6,7]. Therefore, high prices required to produce different regulatory agents used in large quantities limit the use of these agents in animal production. Concurrently, research on QS regulation in specific environments also faces technical challenges related to the specificity of microecosystems. Specifically, the rumen microbiome contains many known and unknown QS signaling molecules, and unknown signaling molecules have the potential to be involved in some important processes, including microbial colonization and optimal fermentation control [87]. A poor grasp of these uncharacterized QS signaling molecules and their associated regulatory networks leaves a crucial knowledge deficit, which acts as a key technical obstacle to the rational design of rumen-specific QS modulators that deliver precise, detectable effects. Safety concerns also persist. The biological toxicity, metabolic fate, and environmental persistence of certain synthetic QSI have not been fully elucidated, sparking legitimate worries about their potential long-term adverse effects on animal physiological functions and ecological safety [88]. While plant-derived QSI are generally considered safer, high-dosage administration may induce a balance of animal gut microbiota or alter feed intake preferences; thus, their therapeutic windows and long-term risks require systematic evaluation [16]. In addition, in comparison with the rather established research framework regarding QSI, the current amount and depth of underlying research and applied exploration regarding QSA is sparse. Thus, the possible security threat and the undetermined risk related to QSA are heightened in relation to that with QSI. Future studies should entail creating species-specific and multi-objective QS modulating agents, optimization of the delivery mechanisms to enhance stability and efficacy and developing standard evaluation systems to ensure safety. By solving the latter challenges QS modulators can transform animal production and present a viable and sustainable alternative to antibiotics and effective heat stress relievers.

## 6. Conclusions

In the scope of the present constraints of QS modulators in livestock production use, future studies are quite necessary, centered on efforts in the following innovation orientations that are based on four main objectives, namely, high targeting efficiency, high stability, low cost and high safety. To begin with, specialized regulators targeting specific QS systems of pathogenic bacteria should be precisely designed based on the specificity of host gut microbiota, physiological metabolic requirements, and specific farming environments. Second, establish a synergistic symbiotic system combining QS modulators with probiotics. Use probiotics to increase the colonization capability as well as the stability of regulatory factors in the gut. At the same time, eliminate the QS systems of the pathogenic bacteria with these regulatory agents, which will also lead to the establishment of favorable ecological niches of the probiotics. Such a synergistic process not only substantially maximizes the regulation of the microecological activity of the gut but also controls the dosage requirements and the risks of resistance to monotherapies. Furthermore, addressing the scalability of diverse QSI and QSA types requires the optimization of extraction, purification, and synthetic pathways to overcome technical barriers to mass production and improve thermal stability. Concurrently, to navigate signal cross-talk within complex microenvironments, priority should be given to developing broad-spectrum, multi-target modulators capable of intercepting multiple QS signaling pathways. In summary, using the specific production of animal-active modulators, the new production of so-called modulator-probiotic compound formulations, the entry of animal programming processes, and the formation of multi-target synergized regulatory networks, we will overcome technological bottlenecks of the moment. This will significantly advance the translational application of QS modulators in animal production, thus providing an essential technological contribution in an effort to attain sustainable livestock development, food safety of animals, and the development of the antibiotic-free farming strategy.

## Figures and Tables

**Table 1:** Naturally occurring quorum sensing inhibitors (QSI) and their inhibitory effects.

QSI	Source	Target	Effect	Reference
Quercetin	Plant extract	Broiler chickens	Reduce inflammation in chicken intestines; enhancing broiler production efficiency; reduce the virulence of pathogenic bacteria	[16]
Vanillin	Plant extract	Broiler chickens	Reduce inflammation in chicken intestines; enhancing broiler production efficiency; reduce the virulence of pathogenic bacteria	[16]
Umbelliferone	Plant extract	Broiler chickens	Reduce inflammation in chicken intestines; enhancing broiler production efficiency; reduce the virulence of pathogenic bacteria	[16]
(-)-*α*-Pinene	Plant extract	*Campylobacter jejuni*	Reduce the virulence of pathogenic bacteria; no adverse effects on broiler production performance	[18]
Chlorogenic acid	Plant extract	Broiler chickens, laying hens, weaned piglets	Reduce heat-induced intestinal damage; reduce the incidence of diarrhea in weaned piglets; enhance antioxidant capacity	[17,19]
Curcumin	Plant extract	Piglets	Alleviate oxidative stress; enhance digestive enzyme activity	[21,22,31]
Resveratrol	Plant extract	Piglets, broiler chickens	Improving the intestinal microenvironment in piglets under heat stress; enhance digestive enzyme activity; enhance antioxidant capacity	[20,21,32]
Paeoniflorin	Plant extract	*Streptococcus suis*	Reduce adhesion virulence; biofilm inhibition; improve survival rates	[23]
Cinnamaldehyde	Plant extract	*Escherichia coli*, chilled pork	Inhibition of *E. coli* AI-2 signaling; Biofilm inhibition; extend shelf life	[24]
Eugenol	Plant extract	*Escherichia coli*, chilled pork	Inhibition of *E. coli* AI-2 signalingBiofilm inhibition; extend shelf life	[24]
Methyl gallate	Plant extract	*Aeromonas hydrophila*	Inhibiting the virulence of *Aeromonas hydrophila;* reduce infection mortality rates	[25]
*Ocimum sanctum*	Plant extract	*Vibrio harveyi*, *Vibrio parahaemolyticus*, *Vibrio vulnificus*	Inhibition of biofilm formation; reduce the secretion of virulence factors	[26]
Artemisinin	Plant extract	Fat greenling (*Hexagrammos otakii*)	Optimize gut microbiota; enhance intestinal mucosal immunity; maintain the integrity of intestinal tissue structure	[27]
Astragalus polysaccharides	Plant extract	Aquatic animals	Alleviate heat stress; reduce inflammatory response; boost immunity	[28]
*Ficus carica* L.	Plant extract	*Chromobacterium violaceum*	Weakening pathogenicity; inhibit the expression of virulence related phenotypes	[29]
*Perilla frutescens*	Plant extract	*Chromobacterium violaceum*	Weakening pathogenicity; inhibit the expression of virulence related phenotypes	[29]
YtnP lactonase	*Bacillus velezensis* D-18	*Vibrio anguillarum*	Inhibition of quorum sensing in *Vibrio anguillarum*; reduce pathogenicity	[33]
AiiA lactonase	*Bacillus thuringiensis*	*Escherichia coli*, *Salmonella*	Improve the production performance of broiler chickens; regulating gut microbiota	[34]
Cyclo(L-Phe-L-Pro)	Microbial metabolite	*Salmonella typhi*	Suppression of virulence and biofilm formation; disorganized injury	[35]
Surfactin	*Bacillus subtilis* 6D1	Multidrug-resistant *Staphylococcus aureus*	Blocking the Agr quorum sensing signaling pathway; enhance antibiotic sensitivity	[36]
2,4-Di-tert-butylphenol (2,4-DTBP)	*Bacillus subtilis* R-18	*Serratia marcescens*	Biofilm inhibition; suppress virulence expression	[37]
Indole metabolites	*Pseudomonas fluorescens*, *Streptomyces thermocarboxydus*	Enterotoxigenic *Escherichia coli*, *Bacillus cereus*	Reduce the virulence of pathogenic bacteria; Destroy the biofilm	[38]
Tannic acid	*Penicillium oxalicum*	*Pseudomonas aeruginosa*	Antitoxic effect; inhibition of toxicity effect; inhibition of biofilm formation	[39]
Ground beef extract	Beef tissue	*Escherichia coli*	Suppression of *E. coli* O157:H7 virulence-associated gene expression	[40]
Ilimaquinone	Sponge extract	*Chromobacterium violaceum*, *Serratia marcescens*, *Pseudomonas aeruginosa*	Anti-biofilm activity; antibiotic alternatives	[41]
Sea cucumber saponins	Sea cucumber extract	*Aeromonas hydrophila*	Inhibition of biofilm formation and virulence expression; reduce pathogenicity; not easy to induce drug resistance	[42]
Peptide SF	*Penaeus vannamei* myosin	*Vibrio parahaemolyticus*	Interfering with bacterial adhesion and aggregation; inhibition of biofilm formation and virulence expression	[43]
Solenopsin A	*Solenopsis invicta* venom	*Pseudomonas aeruginosa*	Suppression of virulence and colonization ability; no risk of inducing drug resistance	[44]
Melittin	Bee venom	*Escherichia coli*, *Staphylococcus aureus*, *Pseudomonas aeruginosa*, *Acinetobacter baumannii*	Possesses triple activity against biofilms, bacteria, and quorum sensing; reduce the risk of developing drug-resistant bacteria; anti biofilm effect	[45]
Cycloartane-type triterpene acids	Propolis extract	*Chromobacterium violaceum*, *Escherichia coli*, *Staphylococcus aureus*, *Pseudomonas aeruginosa*	Inhibits multiple bacteria quorum sensing; antioxidant capacity; inhibition of biofilm formation	[46]

## Data Availability

No new data were created or analyzed in this study. Data sharing is not applicable to this article.

## Abbreviations

The following abbreviations are used in this manuscript:

QS	Quorum sensing
QSI	Quorum sensing inhibitors
AHL	Acyl-homoserine lactone
AI-2	Autoinducer-2
CA	Chlorogenic acid
QSA	Quorum sensing activators

## References

[1] Marshall B.M., Levy S.B. (2011). Food animals and antimicrobials: Impacts on human health. Clin. Microbiol. Rev..

[2] Shini S., Bryden W. (2021). Probiotics and gut health: Linking gut homeostasis and poultry productivity. Anim. Prod. Sci..

[3] Khasanah H., Kusbianto D.E., Purnamasari L., dela Cruz J.F., Widianingrum D.C., Hwang S.G. (2024). Modulation of chicken gut microbiota for enhanced productivity and health: A review. Veter. World.

[4] Sánchez-Jiménez A., Llamas M.A., Marcos-Torres F.J. (2023). Transcriptional regulators controlling virulence in *Pseudomonas aeruginosa*. Int. J. Mol. Sci..

[5] Sakuragi Y., Kolter R. (2007). Quorum-sensing regulation of the biofilm matrix genes (*pel*) of *Pseudomonas aeruginosa*. J. Bacteriol..

[6] Krzyżek P. (2019). Challenges and limitations of anti-quorum sensing therapies. Front. Microbiol..

[7] Kalia V.C. (2013). Quorum sensing inhibitors: An overview. Biotechnol. Adv..

[8] Alum E.U., Gulumbe B.H., Izah S.C., Uti D.E., Aja P.M., Igwenyi I.O., Offor C.E. (2025). Natural product-based inhibitors of quorum sensing: A novel approach to combat antibiotic resistance. Biochem. Biophys. Rep..

[9] Singh A.R. (2018). Regulation of *Streptomyces* chitinases by two-component signal transduction systems and their post translational modifications: A review. J. Pure Appl. Microbiol..

[10] Di Cagno R., De Angelis M., Calasso M., Gobbetti M. (2011). Proteomics of the bacterial cross-talk by quorum sensing. J. Proteomics.

[11] Tavender T.J., Halliday N.M., Hardie K.R., Winzer K. (2008). LuxS-independent formation of AI-2 from ribulose-5-phosphate. BMC Microbiol..

[12] Soto-Aceves M.P., Diggle S.P., Greenberg E.P. (2023). Microbial primer: LuxR-LuxI quorum sensing. Microbiology.

[13] Yu Z., Yu D., Mao Y., Zhang M., Ding M., Zhang J., Wu S., Qiu J., Yin J. (2019). Identification and characterization of a LuxI/R-type quorum sensing system in *Pseudoalteromonas*. Res. Microbiol..

[14] Lee K.-J., Jung Y.-C., Park S.-J., Lee K.-H. (2018). Role of heat shock proteases in quorum-sensing-mediated regulation of biofilm formation by *Vibrio* species. mBio.

[15] Naga N.G., Shaaban M.I. (2023). Quorum sensing and quorum sensing inhibitors of natural origin. Drug Discovery and Design Using Natural Products.

[16] Deryabin D.G., Kosyan D.B., Inchagova K.S., Duskaev G.K. (2023). Plant-derived quorum sensing inhibitors (quercetin, vanillin and umbelliferon) modulate cecal microbiome, reduces inflammation and affect production efficiency in broiler chickens. Microorganisms.

[17] Chen F., Zhang H., Zhao N., Yang X., Du E., Huang S., Guo W., Zhang W., Wei J. (2021). Effect of chlorogenic acid on intestinal inflammation, antioxidant status, and microbial community of young hens challenged with acute heat stress. Anim. Sci. J..

[18] Šimunović K., Sahin O., Kovač J., Shen Z., Klančnik A., Zhang Q., Smole Možina S. (2020). (-)-*α*-Pinene reduces quorum sensing and *Campylobacter jejuni* colonization in broiler chickens. PLoS ONE.

[19] Dong W., Liang G., Gang G., Lu-fang D. (2024). Research progress on role of chlorogenic acid and its application in animal production. Feed Res..

[20] Hong C., Huang Y., Yang G., Wen X., Wang L., Yang X., Gao K., Jiang Z., Xiao H. (2024). Maternal resveratrol improves the intestinal health and weight gain of suckling piglets during high summer temperatures: The involvement of exosome-derived microRNAs and immunoglobin in colostrum. Anim. Nutr..

[21] Wei S., Gan Z., Wei W., Wu J., Xia Z., Chen J., Wang T., Zhong X. (2020). Effects of curcumin and resveratrol combination on digestive enzyme activity and pancreatic antioxidant function in weaned piglets. J. Nanjing Agric. Univ..

[22] Abdulrahman H., Misba L., Ahmad S., Khan A.U. (2019). Curcumin induced photodynamic therapy mediated suppression of quorum sensing pathway of *Pseudomonas aeruginosa*: An approach to inhibit biofilm in vitro. Photodiagn. Photodyn. Ther..

[23] Li J., Shen Y., Zuo J., Gao S., Wang H., Wang Y., Yi L., Hou X., Wang Y. (2022). Inhibitory effect of monoterpenoid glycosides extracts from peony seed meal on *Streptococcus suis* LuxS/AI-2 quorum sensing system and biofilm. Int. J. Environ. Res. Public Health.

[24] Sun X., Wang Z., Zhao X. (2025). Impact of quorum sensing signaling molecule AI-2 and quorum sensing inhibitors on *Escherichia coli* drug resistance and their application in spoilage of chilled pork. Food Biosci..

[25] Jiang H., Wang Z., Jia A. (2024). Methyl gallate from *Camellia nitidissima Chi flowers* reduces quorum sensing related virulence and biofilm formation against *Aeromonas hydrophila*. Biofouling.

[26] Issac Abraham S.V.P., Arumugam V.R., Mary N.I., Dharmadhas J.S., Sundararaj R., Devanesan A.A., Rajamanickam R., Veerapandian R., John Bosco J.P., Danaraj J. (2024). *Ocimum sanctum* as a source of quorum sensing inhibitors to combat antibiotic resistance of human and aquaculture pathogens. Life.

[27] Gu Y., Wang W., Zhan Y., Wei X., Shi Y., Cui D., Peng T., Han J., Li X., Chen Y. (2023). Dietary artemisinin boosts intestinal immunity and healthy in fat greenling (*Hexagrammos otakii*). Front. Immunol..

[28] Bakky M.A.H., Yi P., Tran N.T., Sun Q., Zhang M., Zhang Y., Li S. (2025). Polysaccharide-induced immunoregulation, signaling pathways, and stress mitigation in aquaculture animals: A review. Rev. Aquacult..

[29] Sun S., Li H., Zhou W., Liu A., Zhu H. (2015). Bacterial quorum sensing inhibition activity of the traditional Chinese herbs, *Ficus carica* L. and *Perilla frutescens*. Chemotherapy.

[30] Wells C.W. (2024). Effects of essential oils on economically important characteristics of ruminant species: A comprehensive review. Anim. Nutr..

[31] Aderemi F.A., Alabi O.M. (2023). Turmeric (*Curcuma longa*): An alternative to antibiotics in poultry nutrition. Transl. Anim. Sci..

[32] Kishawy A.T.Y., Ibrahim D.S., Roushdy E.M., Moustafa A., Eldemery F., Hussein E.M., Hassan F.A.M., Elazab S.T., Elabbasy M.T., Kanwal R. (2023). Impact of resveratrol-loaded liposomal nanocarriers on heat-stressed broiler chickens: Effects on performance, sirtuin expression, oxidative stress regulators, and muscle building factors. Front. Vet. Sci..

[33] Monzón-Atienza L., Bravo J., Torrecillas S., Gómez-Mercader A., Montero D., Ramos-Vivas J., Galindo-Villegas J., Acosta F. (2024). An in-depth study on the inhibition of quorum sensing by *Bacillus velezensis* D-18: Its significant impact on *Vibrio* biofilm formation in aquaculture. Microorganisms.

[34] Sun X.X., Chen D.D., Deng S., Zhang G.M., Peng X., Sa R. (2022). Using combined *Lactobacillus* and quorum quenching enzyme supplementation as an antibiotic alternative to improve broiler growth performance, antioxidative status, immune response, and gut microbiota. Poult. Sci..

[35] Jha N.K., Kumar L.L., Sivasankar C., Gopu V., Devi P.B., Murali A., Shetty P.H. (2024). Cyclic di-peptide Cyclo (L-Phe-L-Pro) mitigates the quorum-sensing mediated virulence in *Salmonella typhi* and biofilm formation in poultry and plastic system. Food Biosci..

[36] Leistikow K.R., May D.S., Suh W.S., Vargas Asensio G., Schaenzer A.J., Currie C.R., Hristova K.R. (2024). *Bacillus* subtilis-derived peptides disrupt quorum sensing and biofilm assembly in multidrug-resistant *Staphylococcus aureus*. mSystems.

[37] Devi K.R., Srinivasan S., Ravi A.V. (2018). Inhibition of quorum sensing-mediated virulence in *Serratia marcescens* by *Bacillus subtilis* R-18. Microb. Pathogen..

[38] Rizkinata D., Waturangi D.E., Yulandi A. (2024). Synergistic action of bacteriophage and metabolites of *Pseudomonas fluorescens* JB3B and *Streptomyces thermocarboxydus* 18PM against Enterotoxigenic *Escherichia coli* and *Bacillus cereus* and their biofilm. BMC Microbiol..

[39] Nouh H.S., El-Zawawy N.A., Halawa M., Shalamesh E.M., Ali S.S., Korbecka-Glinka G., Shala A.Y., El-Sapagh S. (2024). Endophytic *Penicillium oxalicum* AUMC 14898 from opuntia ficus-indica: A novel source of tannic acid inhibiting virulence and quorum sensing of extensively drug-resistant *Pseudomonas aeruginosa*. Int. J. Mol. Sci..

[40] Soni K.A., Lu L., Jesudhasan P.R.R., Hume M.E., Pillai S.D. (2008). Influence of autoinducer-2 (AI-2) and beef sample extracts on *E. coli* O157:H7 survival and gene expression of virulence genes *yadK* and *hhA*. J. Food Sci..

[41] Surti M., Patel M., Binsuwaidan R., Adnan M., Alshammari N., Fatima S.B., Reddy M.N. (2025). Ilimaquinone as a novel marine sponge-derived antibacterial agent: Mechanistic insights into its antibiofilm and quorum sensing inhibitory properties targeting bacterial virulence. Int. Microbiol..

[42] Payam B., Soltani M., Mehrgan M.S., Rajabi Islami H., Nazemi M. (2025). Saponins from sea cucumber disrupt *Aeromonas hydrophila* quorum sensing to mitigate pathogenicity. AMB Express.

[43] Yang W., Liu S., Liu X., Yuan S., An L., Ren A., Bai F., Lv X., Li J., Li X. (2025). Novel quorum-sensing inhibitor peptide SF derived from *Penaeus vannamei* myosin inhibits biofilm formation and virulence factors in *Vibrio parahaemolyticus*. LWT.

[44] Park J., Kaufmann G.F., Bowen J.P., Arbiser J.L., Janda K.D. (2008). Solenopsin A, a venom alkaloid from the fire ant *Solenopsis invicta*, inhibits quorum-sensing signaling in *Pseudomonas aeruginosa*. J. Infect. Dis..

[45] Yang H., Ma R., Chen J., Xie Q., Luo W., Sun P., Liu Z., Guo J. (2024). Discovery of melittin as triple-action agent: Broad-spectrum antibacterial, anti-biofilm, and potential anti-quorum sensing activities. Molecules.

[46] Tamfu A.N., Ceylan O., Cârâc G., Talla E., Dinică R.M. (2022). Antibiofilm and anti-quorum sensing potential of cycloartane-type triterpene acids from cameroonian grassland propolis: Phenolic profile and antioxidant activity of crude extract. Molecules.

[47] Sikdar R., Elias M.H. (2020). Quorum quenching enzymes and their effects on virulence, biofilm, and microbiomes: A review of recent advances. Expert Rev. Anti-Infect. Ther..

[48] Song Y., Cai Z., Lao Y., Jin H., Ying K., Lin G., Zhou J. (2018). Antibiofilm activity substances derived from coral symbiotic bacterial extract inhibit biofouling by the model strain *Pseudomonas aeruginosa* PAO1. Microb. Biotechnol..

[49] Zhu H., Liu W., Wang S., Tian B., Zhang S. (2012). Evaluation of anti-quorum-sensing activity of fermentation metabolites from different strains of a medicinal mushroom, *Phellinus igniarius*. Chemotherapy.

[50] Beenker W.A.G., Hoeksma J., den Hertog J. (2022). Gregatins, a group of related fungal secondary metabolites, inhibit aspects of quorum sensing in gram-negative bacteria. Front. Microbiol..

[51] Beenker W.A.G., Hoeksma J., Bannier-Hélaouët M., Clevers H., den Hertog J. (2023). Paecilomycone inhibits quorum sensing in gram-negative bacteria. Microbiol. Spectr..

[52] Zaharioudakis K., Salmas C.E., Andritsos N.D., Kollia E., Leontiou A.A., Karabagias V.K., Karydis-Messinis A., Moschovas D., Zafeiropoulos N.E., Avgeropoulos A. (2024). Carvacrol, citral, eugenol and cinnamaldehyde casein based edible nanoemulsions as novel sustainable active coatings for fresh pork tenderloin meat preservation. Front. Food Sci. Technol..

[53] Wu Y., Yang Y., Zhang Z., Wang Z., Zhao Y., Sun L. (2018). A facile method to prepare size-tunable silver nanoparticles and its antibacterial mechanism. Adv. Powder Technol..

[54] Wang L., Cai Q., Yang Y., Mai Q., Zhou Y., Liu Y., Liu Y., Liu J. (2025). Reshaping bacterial microenvironments: Hybrid biomimetic membrane-coated copper nanosystems combat bacterial biofilm infections by inhibiting bacterial quorum sensing systems. Chem. Eng. J..

[55] Khan M.F., Husain F.M., Zia Q., Ahmad E., Jamal A., Alaidarous M.A., Banawas S.S., Alam M.M., Alshehri B.A., Jameel M. (2020). Anti-quorum sensing and anti-biofilm activity of zinc oxide nanospikes. ACS Omega.

[56] Owrang M., Gholami A. (2024). Green-synthesized silver nanoparticles from *Zataria multiflora* as a promising strategy to target quorum sensing and biofilms in *Pseudomonas aeruginosa*. Heliyon.

[57] Li L., Guan W., Fan Y., He Q., Guo D., Yuan A., Xing Q., Wang Y., Ma Z., Ni J. (2023). Zinc/carbon nanomaterials inhibit antibiotic resistance genes by affecting quorum sensing and microbial community in cattle manure production. Bioresour. Technol..

[58] Olugbojo J.A., Akinyemi A.A., Obasa S.O. (2025). Comparative studies on antibacterial activities of chitosan, silver nanoparticles and maggot based chitosan-silver nanocomposites against fish pathogens. Jordan J. Biol. Sci..

[59] Najafi A., Taheri R.A., Mehdipour M., Martínez-Pastor F., Rouhollahi A.A., Nourani M.R. (2018). Improvement of post-thawed sperm quality in broiler breeder roosters by ellagic acid-loaded liposomes. Poult. Sci..

[60] Gunawan M., Karja N.W.K., Setiadi M.A., Kaiin E.M., Said S., Arifiantini R.I., Iskandar H. (2025). Development and evaluation of soy lecithin-derived nanoliposomes as a plant-based alternative to egg-yolk extender for Ongole-grade bull semen cryopreservation. Vet. World..

[61] Najafi A., Taheri R.A., Mehdipour M., Farnoosh G., Martínez-Pastor F. (2018). Lycopene-loaded nanoliposomes improve the performance of a modified Beltsville extender broiler breeder roosters. Anim. Reprod. Sci..

[62] Besharat M., Islami H.R., Soltani M., Mousavi S.A. (2024). Effects of dietary nanoliposome-coated astaxanthin on haematological parameters, immune responses and the antioxidant status of rainbow trout (*Oncorhynchus mykiss*). Vet. Med. Sci..

[63] de Visser P.J., Karagrigoriou D., Nguindjel A.-D.C., Korevaar P.A. (2024). Quorum sensing in emulsion droplet swarms driven by a surfactant competition system. Adv. Sci..

[64] Helmy Y.A., Kathayat D., Deblais L., Srivastava V., Closs G., Tokarski R.J., Ayinde O.R., Fuchs J.R., Rajashekara G. (2022). Evaluation of novel quorum sensing inhibitors targeting auto-inducer 2 (AI-2) for the control of avian pathogenic *Escherichia coli* infections in chickens. Microbiol. Spectr..

[65] Soni D., Smoum R., Breuer A., Mechoulam R., Steinberg D. (2015). Effect of the synthetic cannabinoid HU-210 on quorum sensing and on the production of quorum sensing-mediated virulence factors by *Vibrio harveyi*. BMC Microbiol..

[66] Zhou J.L., Liu X., Liu Q.X., Liu T.Q., Liu T., Li P.F., Ling F., Wang G.X. (2024). Luteolin-borneol complex, a novel pharmaceutical preparation for aquaculture against NNV infection. Aquaculture.

[67] Defoirdt T., Benneche T., Brackman G., Coenye T., Sorgeloos P., Scheie A.A. (2012). A Quorum sensing-disrupting brominated thiophenone with a promising therapeutic potential to treat luminescent vibriosis. PLoS ONE.

[68] Yan G., Fu L., Ming H., Chen C., Zhou D. (2023). Exploring an efficient and eco-friendly signaling molecule and its quorum quenching ability for controlling microcystis blooms. Environ. Sci. Technol..

[69] Gao S., Shen Y., Yuan S., Quan Y., Li X., Wang Y., Yi L., Wang Y. (2023). Methyl anthranilate deteriorates biofilm structure of *Streptococcus suis* and antagonizes the capsular polysaccharide defence effect. Int. J. Antimicrob. Agents.

[70] Ge J., Li Y., Zhao X., Ouyang K., Qu M., Qiu Q. (2026). Dietary supplementation with D-ribose enhances growth performance, improves serum antioxidant capacity, and inhibits rumen microbial LuxS/AI-2 quorum sensing of Hu sheep. Anim. Biosci..

[71] Liu C., Li L., Dai J., Qu M., Ouyang K., Qiu Q. (2025). Heated drinking water in winter improves growth performance of male Hu sheep by modulating rumen quorum sensing and metabolites, and enhancing serum antioxidant capacity. Anim. Biosci..

[72] Yadav U.R., Devender K., Poornima M., Sekhar C.C., Atcha K.R., Reddy B.S., Padmaja P. (2024). Design, synthesis and biological evaluation of triazole, sulfonamide and sulfonyl urea derivatives of N-acylhomoserine lactone as quorum sensing inhibitors. J. Mol. Struct..

[73] Nayak S.P.R.R., Pohokar P., Das A., Dhivya L.S., Pasupuleti M., Soundharrajan I., Almutairi B.O., Kumaradoss K.M., Arockiaraj J. (2025). Chalcone derivative enhance poultry meat preservation through quorum sensing inhibition against *Salmonella* (*Salmonella enterica* serovar Typhi) contamination. Food Control.

[74] Yeon K.M., Cheong W.-S., Oh H.-S., Lee W.-N., Hwang B.K., Lee C.H., Beyenal H., Lewandowski Z. (2009). Quorum sensing: A new biofouling control paradigm in a membrane bioreactor for advanced wastewater treatment. Environ. Sci. Technol..

[75] Zhang J., Lu K., Zhu L., Li N., Lin D., Cheng Y., Wang M. (2023). Inhibition of quorum sensing serves as an effective strategy to mitigate the risks of human bacterial pathogens in soil. J. Hazard. Mater..

[76] Huang T., Ge H., Wu Z., Zhang Y., Wang L., Dang C., Fu J. (2026). Resistance of microbial community in activated sludge to nano-Ag stress through regulation of N-acyl homoserine lactones-mediated quorum sensing. Biotechnol. Bioeng..

[77] Zha A., Tu R., Qi M., Wang J., Tan B.e., Liao P., Wu C., Yin Y. (2023). Mannan oligosaccharides selenium ameliorates intestinal mucosal barrier, and regulate intestinal microbiota to prevent Enterotoxigenic *Escherichia coli* -induced diarrhea in weaned piglets. Ecotoxicol. Environ. Saf..

[78] Grand E., Respondek F., Martineau C.J., Detilleux J., Bertrand G. (2013). Effects of short-chain fructooligosaccharides on growth performance of preruminant veal calves. J. Dairy Sci..

[79] Fu C., Ge J., Qu M., Ouyang K., Qiu Q. (2025). Effects of 4-hydroxy-2,5-dimethyl-3(2 H)-furanone supplementation on growth performance, serum antioxidant capacity, rumen fermentation characteristics, rumen bacterial quorum sensing, and microbial community in Hu sheep. Anim. Biosci..

[80] Qiu Q., Fu C., Li L., Ouyang K., Qu M. (2026). Dietary 4-hydroxy-2,5-dimethyl-3(2 H)-furanone supplementation enhances serum antioxidant capacity and nutrient digestibility in Hu sheep by promoting biofilm formation. Anim. Feed Sci. Technol..

[81] Liu C., Li L., Li M., Ouyang K., Qu M., Qiu Q. (2026). Dietary bile acids supplementation enhances growth performance, nutrient digestion, hepatic function, rumen fermentation, and modulates quorum sensing in culled ewes. Anim. Feed Sci. Technol..

[82] Liu N., Zhang Y., Zhang Y., Yang Y., Long H., Huang A., Zeng Y., Xie Z. (2025). Quorum sensing mediates spatiotemporal microbial community dynamics and nitrogen metabolism in biofloc-based *Litopenaeus vannamei* aquaculture systems. Bioresour. Technol..

[83] Li Z., Li J., Gong W., Zhang K., Wang G., Xia Y., Yu M., Xie W., Lu Z., Cheng X. (2024). Effect of exogenous acylhomoserine lactone 3-oxo-C14-HSL on the performance of biofilm in moving bed biofilm reactor. J. Water Process Eng..

[84] Yi L., Wu Y., Liu C., Wan L., Han R. (2023). Research progress on quorum sensing enhanced biofilm denitrification performance based on exogenous acyl homoserine lactones. J. Dalian Ocean Univ..

[85] Wang H., Wu P., Zheng D., Deng L., Wang W. (2022). N-Acyl-homoserine lactone (AHL)-mediated microalgal–bacterial communication driving *Chlorella*-activated sludge bacterial biofloc formation. Environ. Sci. Technol..

[86] Bové M., Bao X., Sass A.M., Crabbé A., Coenye T. (2021). The quorum-sensing inhibitor furanone C-30 rapidly loses its tobramycin-potentiating activity against *Pseudomonas aeruginosa* biofilms during experimental evolution. Antimicrob. Agents Chemother..

[87] Lawther K., Santos F.G., Oyama L.B., Huws S.A. (2023). Chemical signalling within the rumen microbiome. Anim. Biosci..

[88] Chen C., Wang D., Wang H., Lin Z., Fang Z. (2017). A SAR-based mechanistic study on the combined toxicities of sulfonamides and quorum sensing inhibitors on *Escherichia coli*. SAR QSAR Environ. Res..

